# The Effect of Adipose-Derived Mesenchymal Stem Cells on Peripheral Nerve Damage in a Rodent Model

**DOI:** 10.3390/jcm12196411

**Published:** 2023-10-09

**Authors:** Mehmet Burak Yalçın, Ejder Saylav Bora, Mümin Alper Erdoğan, Adem Çakır, Oytun Erbaş

**Affiliations:** 1 Department of Orthopedics and Traumatology, Bahcelievler Memorial Hospital, Istanbul 34180, Turkey; mehmetburakyalcin@gmail.com; 2 Department of Emergency Medicine, Izmir Atatürk Research and Training Hospital, Izmir 35360, Turkey; 3 Department of Physiology, Faculty of Medicine, Izmir Kâtip Çelebi University, Izmir 35620, Turkey; alpero86@gmail.com; 4 Department of Emergency Medicine, Çanakkale Mehmet Akif Ersoy State Hospital, Çanakkale 17100, Turkey; dr.ademcakir@hotmail.com; 5 Department of Physiology, Demiroğlu Bilim University, Istanbul 34394, Turkey; oytunerbas2012@gmail.com

**Keywords:** Adipose-derived mesenchymal stem cell, nerve damage, syndecan-1, HSP-70, regeneration

## Abstract

Peripheral nerve damage is a significant clinical problem with limited therapeutic options. Adipose-derived mesenchymal stem cells (ADSCs) have emerged as a promising therapeutic approach due to their regenerative potential. However, the underlying mechanisms by which ADSCs promote peripheral nerve regeneration remain unclear. In this study, we investigated the role of syndecan-1 and heat shock protein 70 (HSP-70) in mediating the regenerative effects of ADSCs on peripheral nerves. ADSCs were characterized and isolated from the adipose tissue of rats. In vitro experiments were conducted to evaluate the ability of ADSCs to secrete syndecan-1 and HSP-70 in response to stress conditions. To evaluate the therapeutic potential of ADSCs, rats with sciatic nerve injuries were treated with ADSCs and assessed for functional recovery, nerve regeneration, and changes in syndecan-1 and HSP-70 levels. Regeneration was evaluated with Electromyography (EMG) histology. The results showed that ADSCs could secrete syndecan-1 and HSP-70 in response to stress conditions. Furthermore, ADSC treatment significantly improved functional recovery and nerve regeneration and increased syndecan-1 and HSP-70 levels in the injured nerve. On the other hand, ADSCs make improvements histologically through the influence of Nerve growth factor (NGF), Malondialdehyde (MDA), and EMG.

## 1. Introduction

Adipose-derived mesenchymal stem cells (ADSCs) are a type of cell used to treat many diseases in recent years [1]. Peripheral nerve damage is a common problem that can result in severe disability and reduced quality of life. It is estimated that approximately 3% of all trauma patients have peripheral nerve injuries. It is reported that there are more than 50 thousand peripheral nerve injuries annually in the United States [2,3]. Current therapies for nerve damage are limited, and there is a need for more effective approaches. Adipose-derived stem cells are a potential treatment option for peripheral neuropathy due to their demonstrated ability to protect and regenerate nerve cells. The literature shows promising results for using ADSCs to treat this condition affecting the peripheral nervous system [4]. When administered to the affected area, ADSCs can differentiate into nerve cells and produce growth factors that promote nerve regeneration and repair [5].

Syndecan-1 is found in high levels in plasma and epithelial cells and has various functions, including regulation of cell migration, proliferation, and differentiation, as well as in inflammation, tissue repair, and wound healing [6]. In nerve injury, studies have demonstrated the involvement of syndecan-1 in regulating tissue repair and inflammation. The release of syndecan-1 from damaged nerve cells is thought to activate immune cells and promote tissue repair [7]. In addition, studies in the literature show that it increases neurogenesis in nerve tissue after injury [8].

Heat shock protein 70 (HSP-70) is a type of molecular chaperone, which is a protein that assists in the folding and assembly of other proteins. HSP-70 is a family of heat shock proteins synthesized in response to various cellular stressors, including high temperature, oxidative stress, and inflammation [9]. It has also been shown to have neuroprotective effects in various nerve injury models, such as spinal cord injury and neuropathic pain. It may help prevent cell death and promote cell survival by preventing protein misfolding and facilitating the repair of damaged proteins [10].

One of the parameters through which ADSCs exert their therapeutic effects involves upregulating the transmembrane proteins syndecan-1 and HSP-70. These parameters promote nerve regeneration by enhancing cell survival, reducing inflammation, and stimulating axonal growth. Understanding the mechanisms of ADSC-mediated nerve regeneration through the syndecan-1 and HSP-70 parameters may lead to the development of novel therapeutic strategies for peripheral nerve damage [11].

This study was conducted only with rats to evaluate the curative effect of ADSCs implantation in the sciatic nerve injury model performed with rats in terms of syndecan-1, HSP-70, and nerve growth factor (NGF) levels with electrophysiological and histopathological methods. In our study, ADSC treatment was used in peripheral nerve injuries for which no clear treatment has been established, and the effects of the mediators or pathways on peripheral nerve injury were investigated and presented.

## 2. Materials and Methods

### 2.1. Animals

The research involved 30 adults male Wistar rats weighing an average of 205 g each. The rats were kept in a controlled environment with a room temperature maintained at 22 ± 2 °C and a light-dark cycle of 12 h each. They were given a standard pellet diet and allowed unrestricted access to tap water throughout the experiment. All experimental protocols were approved by an Animal Care and Ethical Committee of the Demiroğlu Science University institutional and licensing committee with ethical number 042340917. Sigma-Aldrich Inc. supplied all the chemicals utilized in the research, except in cases where other sources were explicitly mentioned. The experiments performed in this study have been carried out according to the rules in the Guide for the Care and Use of Laboratory Animals adopted by the National Institutes of Health (Bethesda, MD, USA). All methods are reported following ARRIVE guidelines.

### 2.2. Experimental Protocol

The experiment consisted of 30 male Wistar rats, of which 20 underwent a surgical procedure to expose and repair their sciatic nerves and were assigned to the experimental group. The remaining ten rats were assigned as the control group and underwent no surgical or drug treatment. Within the experimental group, there were two subgroups: the surgery and saline (S&S) group (*n* = 10) received an intraperitoneal injection of 1 mL/kg 0.9% sodium chloride (NaCl) as a placebo treatment. In contrast, the surgery and mesenchymal stem cells [(MSC) (S&M)] group (*n* = 10) received an intraperitoneal injection of 2.0 × 10^6^ cells/kg ADSCs twice a week for 12 weeks, resulting in a total dose of 48 × 10^6^ cells/kg. Following the 12-week administration of the drug, a motor function test was performed, and electromyography (EMG) recordings were obtained.

At the end of the experiment, all rats were given anesthesia (100 mg/kg Ketasol, Richterpharma AG, Wels, Austria) and xylazine (50 mg/kg Rompun, Bayer, Berlin, Germany) before being euthanized by cervical dislocation. Blood samples were collected through cardiac puncture for biochemical analysis, and sciatic nerve samples were collected for immunohistochemistry and biochemical analysis.

### 2.3. Isolation of Mesenchymal Stem Cells from Adipose Tissue

The mesenchymal stem cells (Stembio Cell and Tissue Technologies Inc., Istanbul, Turkey) were obtained from the adipose tissue around the rats’ flanks. The rats were anesthetized using a combination of Ketasol (50 mg/kg) from Richterpharma AG, Austria, and xylazine (10 mg/kg) from Bayer, Germany, to facilitate the procedure. Under sterile conditions, the adipose tissue was carefully removed and transported on ice to the stem cell laboratory. The tissue was then cut into small pieces and placed in a 0.2% collagenase type II solution from Gibco, Grand Island, NY, USA. The mixture was subjected to continuous shaking at 37 °C for 40 min. Following centrifugation, the resulting pellet was suspended in 2 mL of Dulbecco’s Modified Eagles Medium (DMEM, Gibco, USA) and added to culture flasks containing 3 mL of DMEM supplemented with 10% fetal bovine serum (FBS, Gibco, USA), 1% penicillin, 1% streptomycin, and two mM L-glutamine. The flasks were then transferred to an incubator with 5% CO_2_ at 37 °C and saturated humidity. After three days, the growth medium was replaced to ensure the cells reached 85% confluence. The cells were subcultured and passaged up to the fourth passage using 0.25% trypsin (Gibco, USA). The trypsin activity was stopped by adding an equal volume of DMEM to the culture. The ADSCs obtained from the fourth passage were preserved by cryopreservation for their future use in cell transplantation. To accomplish this, 2 × 10^6^ viable cells/mL were suspended in a solution containing 50% DMEM, 40% FBS, and 10% dimethyl sulfoxide (DMSO; MP Bio, Irvine, CA, USA) and stored in labeled cryovials. The cryovials were kept in a nitrogen tank at −196 °C. The cells were monitored for their morphology and growth using an inverted microscope. When required for transplantation, the cells were thawed by immersing the cryovials in a water bath at 37 °C. Next, the cells were centrifuged for 5 min at 1500 rpm, and the pellet obtained was re-suspended in DMEM. The cells were then incubated in a CO_2_ incubator with a temperature of 37 °C and saturated humidity until they were ready for transplantation.

### 2.4. Mesenchymal Stem Cell Characterization

In the second passage of ADSCs, their properties were evaluated by performing immunofluorescence staining. The cells were cultivated in dishes, washed with phosphate-buffered saline (PBS), and then treated with methanol for 5 min at −10 °C to fix them. Normal goat serum was used for 20 min before washing them with PBS to block the cells. Next, primary antibodies targeting CD13, CD29, and CD105 molecules were added to the cells and incubated for 1 h. After washing with PBS for 5 min, a secondary antibody was added and incubated for 45 min. The cells were then rewashed with PBS.

The researchers confirmed the purity and identity of the isolated ADSC population by performing immunofluorescence staining at room temperature. The cells were mounted with a mounting medium and viewed under a fluorescence microscope. This technique confirmed the expression of specific markers for ADSCs, including CD13, CD29, and CD105 molecules. The use of these markers is crucial for the accurate identification of ADSCs.

### 2.5. Surgical Procedure

During the experiment, the rats received a general anesthetic comprising ketamine and xylazine at 75 mg/kg and 10 g/kg, respectively. They were then placed in the prone position on an operating table, and the aseptic technique was used to expose their sciatic nerves. The exposure was carried out from 1 cm distal to the sciatic notch to 1 cm distal to the point where the nerve divides into three branches. A nerve segment of 3–3.5 cm in length, located above the point where the sciatic nerve divides into three branches, was isolated. Using microscissors, the nerve was cut at a distance of 1.5 cm from the trifurcation point. The transected nerve was repaired using Ethilon^®^ 9-0, and the same surgeon placed three epineural sutures. The wound was then closed using a 3-0 Vicryl^®^. Following the procedure, the rats were allowed to regain consciousness and were provided with food and water. They were kept under observation during recovery.

### 2.6. Electrophysiological Recordings

**Figure 1:**
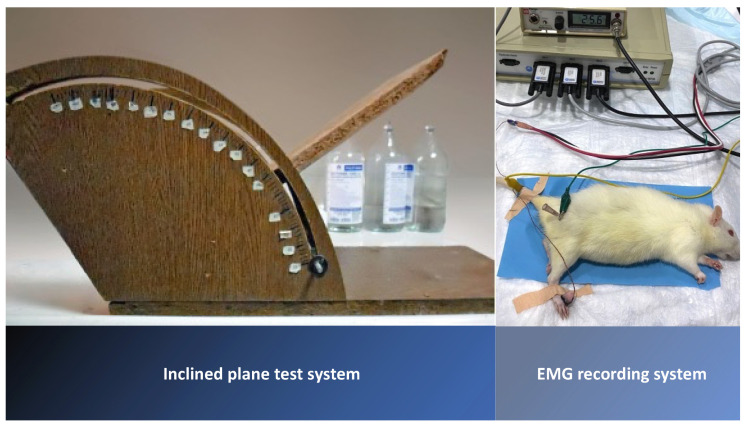
Inclined plane test system and EMG recording system.

To anesthetize the rats in this study, a combination of ketamine hydrochloride (Alfamine, Alfasan International BV Holland, Woerden, The Netherlands) at a dosage of 80 mg/kg and xylazine hydrochloride (Alfazyne, Alfasan International BV Holland) at a dosage of 10 mg/kg was administered. At the end of the experiment, electrophysiological recordings, specifically EMG studies, were performed. To stimulate the right and left sciatic nerves, a bipolar subcutaneous needle stimulation electrode (BIOPAC Systems, Inc., Santa Barbara, CA, USA) was used, and supramaximal stimulation was applied from the sciatic notch. The EMG was performed three times by applying supramaximal stimulation at 10 V with a duration of 0.05 ms and a frequency of 1 Hz. The recordings were taken in the frequency range of 0.5–5000 Hz at a sampling rate of 40 kHz/s (Figure 1). Compound muscle action potentials (CMAPs) were recorded from 2–3 interosseous muscles using unipolar platinum electrodes. The data were analyzed using Biopac Student Lab Pro version 3.6.7 software from BIOPAC Systems, Inc., Goleta, CA, USA. The distal latency and amplitude of CMAP were the parameters used to analyze the data. During the EMG recordings, rectal temperatures of the rats were monitored using a rectal probe (HP Viridia 24-C; Hewlett-Packard Company, Palo Alto, CA, USA), and a heating pad was used to maintain a temperature of approximately 36–37 °C for each rat. The experiments were conducted between 10:00 a.m. and 2:00 p.m.

### 2.7. Assessment of Motor Function via Inclined Plane Test

An inclined-plate test was conducted to evaluate the rats’ motor abilities. The rats were placed obliquely on the long axis of the plate, which had an initial angle of 10 degrees. The angle of the plate was then gradually increased until the rat could maintain its position for 5 s without falling. The angle at which the rat could maintain its position for 5 s was recorded as the motor score. The inclined plate angle was measured three times for each rat, and the average value was calculated (Figure 1).

### 2.8. Histology and Quantitative Histochemistry

For histological and quantitative immunohistochemical assessments, the sciatic nerves of the rats were perfused with a 4% formaldehyde solution through the intracardial route. The distal portions of the sciatic nerves, approximately 10 mm away from the transected site, were extracted, embedded in paraffin, and cut into sections that were 5 µm thick using a microtome (Leica RM 2145, Nussloch, Germany). Hematoxylin and eosin (H&E) staining was utilized to label the axons.

To assess the level of fibrosis, the damaged cells in at least five randomly chosen regions were counted. The percentage of fibrosis was then calculated by dividing the total number of cells in the area by the number of cells counted.

The endogenous peroxidase activity was neutralized by treating the samples with 10% hydrogen peroxide for 30 min to prepare the tissue samples for immunohistochemical analysis. Next, normal goat serum was applied to block non-specific binding sites, followed by incubation with a specific primary antibody against Nerve growth factor (NGF) (Santacruz Biotechnology, diluted 1/100) for 24 h at 4 °C. The antibodies were then detected using the Histostain-Plus Bulk kit, which is specific to rabbit immunoglobulin G, and the resulting signal was visualized using 3,3′-diaminobenzidine (DAB). The stained samples were evaluated to assess the expression of NGF.

The samples were washed with PBS and examined under an Olympus BX51 microscope. Images were captured using an Olympus C-5050 camera. All groups were subjected to quantitative immunohistochemistry analysis, and six slices from each rat were evaluated. Two evaluators blinded to the experimental conditions performed the counting of Schwann cells and axons that exhibited immune-positive staining. They used a light microscope with various magnifications of 20×. The data were presented as the mean ± standard error of the mean.

This study measured two parameters: the quantity and size of axons. Six randomly chosen fields, consisting of one central and five peripheral areas, were analyzed at a magnification of 20×. To obtain cross-sections, a digital counter was used with a grid.

### 2.9. Nerve Biochemical Analysis

The sciatic nerves of the rats were removed after their deaths and stored at a temperature of −20 °C for subsequent biochemical analysis. The distal parts of the nerves, which were approximately 10 mm away from the site of transection, were crushed using a glass homogenizer along with PBS saline with a pH of 7.4 at a ratio of 5:1. The homogenates were subjected to centrifugation at 5000× *g* for 15 min, and the supernatant was collected. Bradford’s method and bovine serum albumin as a reference standard

### 2.10. Determination of the Total Protein Concentration in the Supernatant

To determine the number of specific proteins in the nerve homogenate samples, (Enzyme-Linked Immunosorbent Assay (ELISA) was employed. Syndecan-1, NGF, and HSP-70 levels were quantified using commercially available rat ELISA kits. The ELISA assay uses antibodies immobilized onto a microplate to bind to the protein of interest in the sample specifically. After removing any unbound substances, a secondary antibody conjugated to an enzyme is added to the wells. The protein samples are added to wells containing specific antibodies immobilized on a microplate during the ELISA assay. The proteins of interest bind to the immobilized antibodies, and any unbound substances are removed. Then, a secondary antibody that is attached to an enzyme is introduced to the wells. The enzyme catalyzes a reaction with a substrate added to the wells, which produces a detectable signal. The strength of the signal is proportional to the amount of protein in the sample. A microplate reader (Thermo Scientific Multiskan GO, Fisher Scientific Oy Ratastie Finland) is used to measure the signal’s absorbance, and a standard curve is generated using known protein concentrations. The protein concentration in the sample is then determined based on the standard curve.

### 2.11. Statistical Analysis

The data were analyzed with SPSS Package Program version 26.0. Number, percentage, mean, standard deviation, median, minimum, and maximum were used in the presentation of descriptive data. The conformity of the data to the normal distribution was evaluated with the Kolmogorov-Smirnov Test. Parametric variables between groups were compared using the Student’s *t*-test. The results were reported as mean ± standard deviation (SD) for each group, and multiple comparisons were examined using one-way ANOVA. For the one-way ANOVA test, the Bonferroni type was chosen. A *p*-value of less than 0.05 was considered statistically significant, whereas a *p*-value of less than 0.001 was considered highly effective.

## 3. Results

After the procedure on sciatic nerve electromyographic compound muscle action potential (EMG CMAP) latency, the S&S group became statistically longer (3.48 ± 0.07 ms) (*p* < 0.01) than the normal group (2.32 ± 0.09 ms). Between the S&S group and the S&M group, no statistical difference was observed.

Moreover, in EMG and CMAP, the amplitude of the normal group (15.8 ± 1.2 mV) is statistically higher (*p* < 0.01) than the S&S group (2.2 ± 0.1 mV), and after ADSCs, the S&M group amplitude is statistically higher (*p* < 0.01) (9.8 ± 0.5 mV) than the S&S group (2.2 ± 0.1 mV).

**Figure 2:**
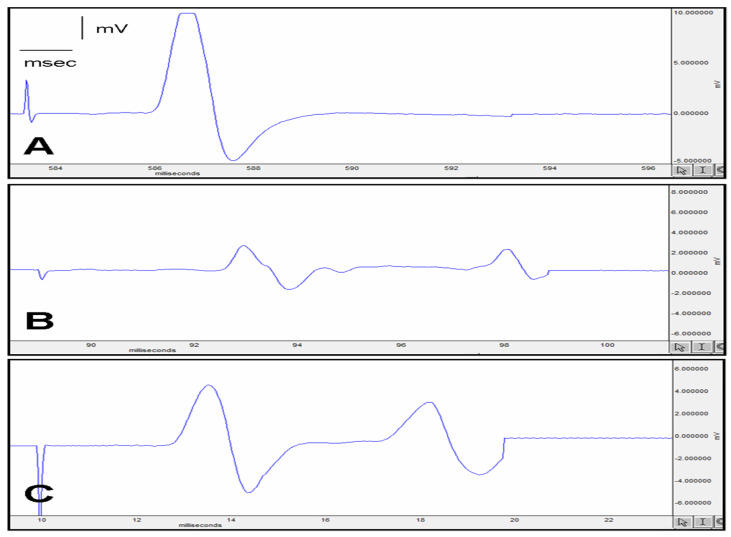
(**A**) Control group EMG; (**B**) Surgery and Saline Group EMG; (**C**) Surgery and MSC group EMG.

Inclined plane score (°) decreased statistically in the S&S (30.5 ± 5.9°) (*p* < 0.01) group than the normal group (85.2 ± 3.3°) and increased after the ADSCs procedure in the S&M group (81.2 ± 4.7°) (*p* < 0.01) than the S&S group (30.5 ± 5.9°) (Figure 2).

These results show that although the administration of ADSCs increased CMAP latency compared to the S&S group, it significantly increased the CMAP amplitude compared to the S&S group and significantly improved nerve action potential. Again, it is seen that the inclined plane score (°) is brought to the level of the control group (Figure 2).

**Figure 3:**
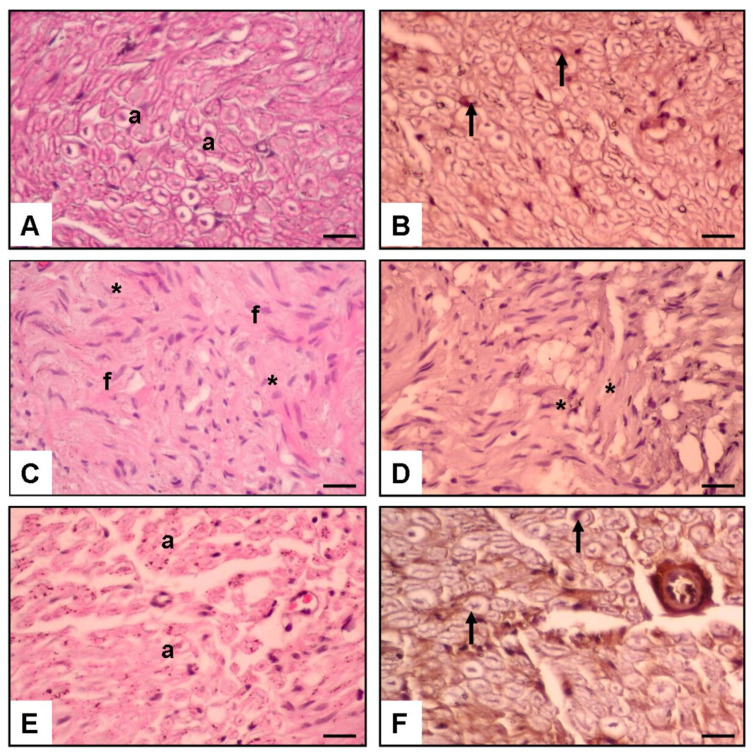
Hematoxylin, and Eosine and NGF immunostainingimmunostaining. (**A**,**B**): Normal Control Group Normal axons (a) and Schwann cells (arrow). (**C**,**D**): Surgery and Saline Group. Increased fibrosis (f), diminished axon, Schwann cell, and NGF immunoexpression (asterisk). (**E**,**F**): Surgery and MSC Group increased axon (a), schwann cells, and NGF immunexpression (arrow) (×20 Magnification) (scale bar = 200 μm).

Figure 3 shows three panels: (A–F) The first panel (A,B) represents the normal control group and shows a normal axon with a visible Schwann cell (indicated by arrows). The second panel (C,D) represents the S&S group and shows increased fibrosis (indicated by f) and a significantly diminished axon, Schwann cell, and NGF immunoexpression (indicated by an asterisk). The third panel (E,F) represents the S&M group and shows increased axon (indicated by a), Schwann cell, and NGF immunoexpression (indicated by an arrow).

Histological images of a nerve under ×20 magnification. The nerve is stained with H&E, which allows visualization of the axons and surrounding tissues. Additionally, Figure 3 shows NGF immunostaining, which indicates the presence of NGF protein in the nerve tissue.

The normal control group had a mean value of 86.2 ± 2.9 for NGF immunoexpression on Schwann cells. The S&S group had a significantly lower mean value of 4.4 ± 0.2 (*p* < 0.01), while the S&M group had a mean value of 78.6 ± 6.1 (*p* < 0.01).

The normal control group had a mean value of 312.9 ± 12.1 for the total axon number. The S&S group had a significantly lower mean value of 19.8 ± 3.7 (*p* < 0.01), while the S&M group had a mean value of 215.5 ± 10.6 (*p* < 0.001).

The normal control group had a mean value of 3.55 ± 0.2 for axon diameter. The S&S group had a significantly lower mean value of 1.66 ± 0.3 (*p* < 0.05), while the S&M group had a mean value of 3.39 ± 0.2 (*p* < 0.001).

**Figure 4:**
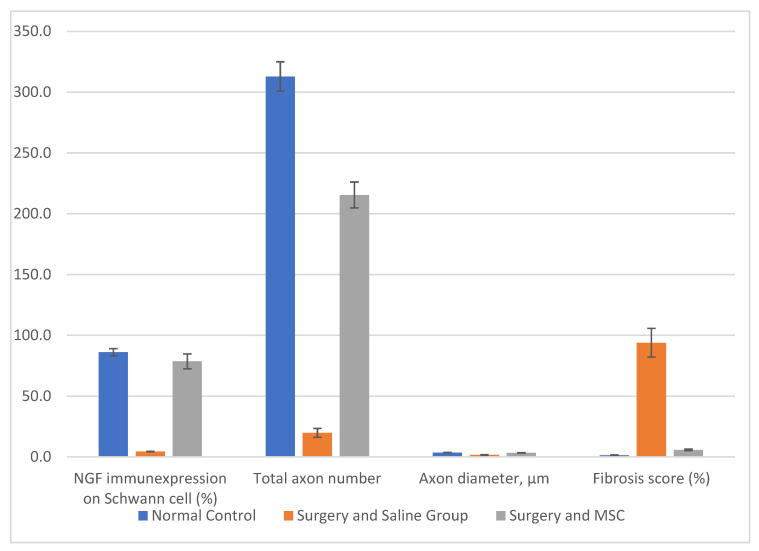
NGF immunoexpression on Schwann cells, total axon number, axon diameter, and fibrosis score.

For the fibrosis score, the normal control group had a mean value of 1.3 ± 0.5. The S&S group had a significantly higher mean value of 93.9 ± 11.8 (*p* < 0.01), while the S&M group had a mean value of 5.8 ± 0.6 (*p* < 0.001). These results indicate that NGF enhances the repair and renewal of Schwann cells while reducing fibrosis (Figure 4).

The normal control group had a mean MDA level of 96.8 ± 5.5. The S&S group had a significantly higher mean value of 175.4 ± 6.8 (*p* < 0.05), indicating increased oxidative stress. On the other hand, the S&M group had a mean MDA value of 102.3 ± 7.7 (*p* < 0.05), which was not significantly different from the control group.

For the nerve syndecan-1 level, the normal control group had a mean value of 16.5 ± 4.6. The S&S group had a mean value of 18.5 ± 2.9, which was not significantly different from the control group. The S&M group had a significantly higher mean value of 44.1 ± 5.2 (*p* < 0.05).

This indicates that the average NGF level in the normal control group was 22.9 units, with a standard deviation of 0.8. The group that underwent surgery and received saline treatment had a significantly lower mean NGF level of 11.7 ± 1.5 compared to the normal control group (*p* < 0.01). On the other hand, the S&M group had a significantly higher mean NGF level of 34.6 ± 3.09 compared to the normal control and S&S groups (*p* < 0.01 in both groups).

The mean value of the nerve HSP-70 level in the normal control group was 6.11 ± 0.7. The S&S group had a mean value of 7.05 ± 1.3, which was not significantly different from the control group. On the other hand, the S&M group had a significantly higher mean value of 20.9 ± 2.5 (*p* < 0.01).

**Figure 5:**
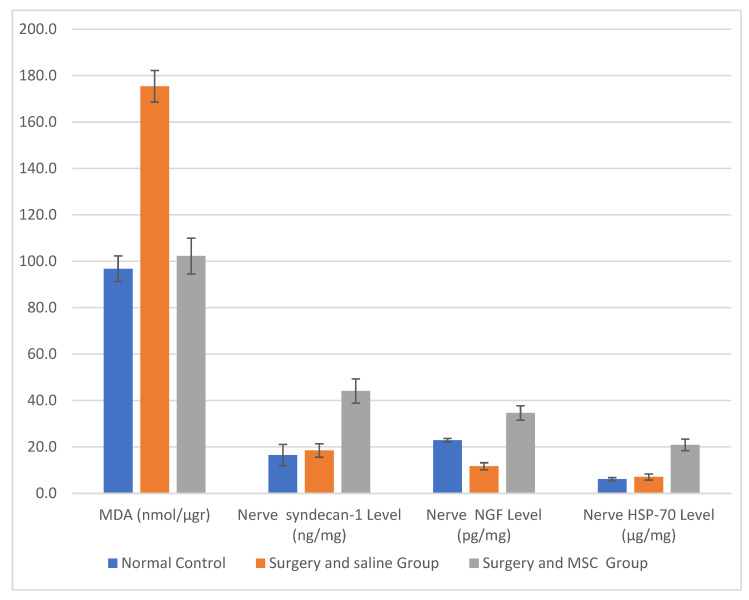
Malondialdehyde (MDA) level, nerve syndecan-1 level, NGF level, and nerve HSP-70 level (these data were obtained by ELISA).

These results show us that ADSCs reduce MDA levels; on the other hand, ADSCs appear to increase syndecan-1, NGF, and HSP-70 levels. This shows that the increase exceeds normal control group levels (Figure 5).

Considering all these results, our results show that ADSCs provide significant positive benefits to the healing process of peripheral nerve injuries, thanks to the mediators that ADSCs affect and the parameters that they activate/inhibit, and that they are promising in the treatment of peripheral nerve injuries.

## 4. Discussion

### 4.1. Why Do We Use ADSCs?

The regeneration of the sciatic nerve after injury is a significant challenge in regenerative medicine. Adipose-derived mesenchymal stem cells have been investigated as a potential therapy for sciatic nerve regeneration [12]. In a recent study, the effects of ADSC transplantation on sciatic nerve regeneration were evaluated by measuring syndecan-1, HSP-70, MDA, and NGF levels of the nerve, as well as by evaluating the electroencephalogram (EEG) of the sciatic nerve.

On the other hand, there are studies that give hope for curing with stem cells, not only in peripheric nerve injury but in central nerve injuries such as stroke [13].

### 4.2. NGF Expression

Several studies have investigated the effects of ADSCs on NGF expression in Schwann cells after nerve injury. In a study by Zhang et al. [10], ADSCs were implanted into the sciatic nerve of rats following injury, resulting in an increase in NGF expression in Schwann cells compared to the injury-only group. Similarly, in a study by Ran et al. [14], ADSCs were shown to upregulate NGF expression in Schwann cells and promote nerve regeneration after sciatic nerve injury. Similarly, in our study, NGF expression increased after ADSC implantation in sciatic nerve-damaged rats.

In another study, Liu et al. [15] demonstrated that the combination of ADSCs and NGF treatment resulted in significantly improved functional recovery and nerve regeneration compared to either treatment alone. These findings suggest that using ADSCs in combination with NGF may be a promising strategy for promoting nerve regeneration following injury.

### 4.3. Syndecan-1 and HSP-70 Expression

Studies have shown that syndecan-1 expression is upregulated in response to nerve injury and is involved in the recruitment and activation of immune cells at the injury site [16]. Syndecan-1 has been shown to play a role in the activation of macrophages, which are critical for the clearance of debris and the promotion of nerve regeneration [17]. Furthermore, syndecan-1 has been found to regulate the expression of growth factors and extracellular matrix components that are important for nerve regeneration. For example, syndecan-1 has been shown to upregulate the expression of NGF and brain-derived neurotrophic factor (BDNF), which are critical for neurons’ survival and growth [18]. This study shows that syndecan-1 augmentation decreases the fibrosis percentage of nerves after injury through the cleaning properties of syndecan-1; on the other hand, syndecan-1 provides healing by promoting NGF expression in the injured area.

In a study by Erbas and his colleagues [19], the levels of syndecan-1 and HSP-70 were measured in rats with sciatic nerve injuries before and after the implantation of ADSCs. The results showed that while the levels of syndecan-1 and HSP-70 did not change significantly after nerve injury, they increased significantly following ADSC implantation. These findings suggest that ADSCs promote nerve regeneration and repair by upregulating the expression of syndecan-1 and HSP-70.

Several other studies have also investigated the effects of ADSCs on the expression of syndecan-1 and HSP-70 in various tissues [9,20]. In a study by Chang et al. [21], ADSCs were shown to upregulate the expression of HSP-70 in ischemic myocardium, leading to improved cardiac function. Similarly, in a study by Matthay et al. [22], ADSCs were shown to increase the expression of syndecan-1 and HSP-70 in rats with acute lung injury, leading to reduced inflammation and improved lung function.

The mechanisms underlying the effects of ADSCs on the expression of syndecan-1 and HSP-70 still need to be fully understood. However, it has been suggested that ADSCs may promote the activation of various signaling pathways, such as the PI3K/Akt and MAPK/ERK pathways, which regulate syndecan-1 and HSP-70 expression [23]. However, in general, the characteristics and biological functions of the PI3K/AKT and MAPK/ERK pathways are as follows: they are potential stem cell signaling pathways that refer to the regulation of stem cell self-renewal, differentiation, and proliferation [24].

### 4.4. MDA Effect in Nerve Damage

As we know, HSP-70 and NGF are co-workers, and they need each other to be secreted in a case of nerve injury for forced regeneration. As a proteoglycan, syndecan-1 can help nerve cells migrate from the injured area and repair the damaged area. Of all these markers, MDA has a different effect after nerve damage; as we know, MDA can cause cell damage by causing the peroxidation of lipids in nerve cells [25]. Therefore, MDA may negatively affect the regeneration process after nerve damage and inhibit the healthy regeneration of nerve cells. Many studies have tried many antioxidants to prevent damage to MDA [26,27]. In this study, ADSCs, other than antioxidants, prevent peroxidation and protect against nerve damage by decreasing MDA levels.

### 4.5. Effect of ADSCs on Nerve Axon

Another important finding of this study is the effect of ADSCs on the total axon number in the injured nerve. The S&S group showed a significant decrease in total axon number compared to the normal control group. In contrast, the S&M group had a mean total axon number significantly higher than the S&S group. This finding suggests that ADSCs can promote axonal regeneration after nerve injury, which is consistent with previous studies that have shown the potential of ADSCs to enhance axonal growth and improve nerve function [28,29].

Furthermore, ADSCs were found to have a positive effect on axon diameter. The S&S group showed a significant decrease in axon diameter compared to the normal control group. In contrast, the S&M group had a mean value of axon diameter that was close to the normal control group. This finding suggests that ADSCs can also promote axonal myelination and improve nerve function, consistent with previous studies that have demonstrated the potential of ADSCs to promote remyelination and prevent fibrosis in damaged nerves [30,31].

### 4.6. Effect of ADSC on Axon Conduction

In EMG, CMAP amplitude increased in the ADSCs group when compared with the saline group; on the other hand, CMAP latency did not have a statistical difference in the saline group and ADSCs group. From these findings, the nerve’s CMAP latency cannot change quickly after injury if we compare it with the CMAP compound. The inclined plane score, which we can also use to provide muscle strength, shows that after ADSC implantation, the degree of the plane becomes almost like before the sciatic injury. This result is similar to the CMAP compound results.

These findings are consistent with previous studies that have demonstrated the potential of ADSC implantation for promoting nerve regeneration and functional recovery after peripheral nerve injury [32,33,34]. ADSCs are a promising source of stem cells for nerve regeneration due to their high proliferation rate, multipotency, and immunomodulatory effects. Previous studies have shown that ADSCs can enhance the expression of growth factors, including NGF and BDNF, essential for nerve regeneration and functional recovery [35,36].

Considering all these results, there is a significant difference between the recovery rate achieved when patients with nerve damage are left to the normal healing process and the use of ADSCs in treatment. This provides us with a useful treatment for nerve damage repair using ADSCs and other explainable mechanisms and signaling pathways. Future studies on this subject suggest that it will reduce morbidity and eliminate the resulting economic and workforce loss in patients with nerve damage where treatment cannot be provided at a significant level. In addition, it produces satisfactory results for patients who move away from the social environment due to morbidity.

### 4.7. Limitations

Our study has several limitations. One of these limitations is that the ADSCs we use in nerve repair can trigger or inhibit different pathways for nerve repair that we have not been able to detect. Since there is not much data in the literature on this subject, the pathways or mechanisms that ADSCs inhibit or activate in tissues are still not fully clarified. We think that there may be another mechanism that activates or inhibits in repairing nerve damage through unexplained mechanisms. However, these pathways or mediators will not significantly change our study results. The results show that ADSCs can alter the levels of MDA, syndecan-1, HSP70, and NGF; however, the data are insufficient to describe how ADSCs mitigate peripheral nerve damage. Further studies will show us the exact mechanism for this subject.

## 5. Conclusions

According to the results of this study, ADSC implantation in sciatic nerve damage significantly contributes to the realization and acceleration of nerve regeneration. Further studies will illuminate the details of this subject. And, investigating the effect of the combined use of mediators or pathways identified in the treatment of peripheral nerve injury on the treatment will lead to maximum results from the treatment. For this reason, this situation should be taken into consideration in future studies.

## Data Availability

All the data for this study are presented in the published article. Upon reasonable request, any further details are available from the corresponding author (Ejder Saylav Bora, saylavbora@hotmail.com).
